# Spatio-temporal transformer traffic prediction network based on multi-level causal attention

**DOI:** 10.1371/journal.pone.0331139

**Published:** 2025-09-02

**Authors:** Hengyuan He, Zhengtao Long, Yingchao Zhang, Xiaofei Jiang

**Affiliations:** College of Big Data and Information Engineering, GuiZhou University, Guiyang, Guizhou, China; National University of Defense Technology, CHINA

## Abstract

Traffic prediction is a core technology in intelligent transportation systems with broad application prospects. However, traffic flow data exhibits complex characteristics across both temporal and spatial dimensions, posing challenges for accurate prediction. In this paper, we propose a spatiotemporal Transformer network based on multi-level causal attention (MLCAFormer). We design a multi-level temporal causal attention mechanism that captures complex long- and short-term dependencies from local to global through a hierarchical architecture while strictly adhering to temporal causality. We also present a node-identity-aware spatial attention mechanism, which enhances the model’s ability to distinguish nodes and learn spatial correlations by assigning a unique identity embedding to each node. Moreover, our model integrates several input features, including original traffic flow data, cyclical patterns, and collaborative spatio-temporal embedding. Comprehensive tests on four real-world traffic datasets—METR-LA, PEMS-BAY, PEMS04, and PEMS08—show that our proposed MLCAFormer outperforms current benchmark models.

## Introduction

Traffic flow prediction is becoming an essential component of developing intelligent transportation systems (ITS) due to the fast urbanization and growing complexity of transportation networks. By integrating historical data and using advanced prediction algorithms, traffic management authorities can dynamically optimize resource scheduling, reduce traffic jams, and thus improve overall traffic efficiency [1]. Currently, a large number of sensors have been deployed on urban roads, which also provide rich data support for traffic prediction [2]. Furthermore, the primary obstacle in traffic flow prediction is the efficient modeling and integration of the spatiotemporal relationships of urban traffic networks [3].

In recent years, traffic prediction methods have changed all the time, showing that we are learning more about the complex spatial and temporal aspects of this field. Researchers in the past used standard statistical models, like the Autoregressive Integrated Moving Average (ARIMA) model [4], to do this job. These models perform well with stationary and linear data but tend to perform poorly when faced with non-linear and dynamic data. Subsequently, the introduction of machine learning methods, such as KNN [5], overcame this issue to some extent. However, they require tedious feature engineering and failed to consider the spatial dependencies within the data.

The rapid development of deep learning has significantly advanced research in the field of traffic prediction. Initial explorations combined Convolutional Neural Networks (CNNs) for extracting spatial features with Recurrent Neural Networks (RNNs) for modeling temporal dependencies. As research has deepened, Graph Neural Networks (GNNs) [6] have emerged, showcasing immense potential due to their powerful capabilities in processing network-structured data, with the STGCN model [7] being a successful example. Recently, the Transformer architecture [8] has shown great promise in traffic forecasting, its core advantages being the ability to perform parallel data processing and its proficiency in modeling long-term dependencies. This technological progress has facilitated the development of advanced models like GMAN [9] and PDFormer [10].

However, despite the significant progress made by existing methods, the following key challenges remain in addressing the complex dynamics of real-world traffic systems:

(1) **Multi-scale and causality challenges in time series modeling.** When applying the standard self-attention mechanism to time-series forecasting, a core challenge is the effective capture of both long- and short-term dependencies. Traffic flow data, for example, naturally contains complex patterns ranging from local fluctuations to medium- and long-term trends. However, mainstream attention-based models [9–11] employ fixed receptive fields for temporal processing, limiting their ability to capture and integrate multi-granularity features effectively. Another critical issue is ensuring temporal causality during multi-scale modeling. This principle dictates that future states must not influence inferences about current or past states, so as to prevent information leakage. Therefore, designing a temporal attention mechanism that can efficiently capture long- and short-term dependencies while strictly adhering to the law of causality is key to enhancing the performance and reliability of prediction models.
(2) **Existing limitations in spatial relationship modeling.** On the one hand, while Graph Neural Networks (GNNs) can effectively learn spatial dependencies between nodes, their performance is highly dependent on a predefined, static adjacency matrix [7,12]. This makes it challenging for the model to capture spatial dependencies that evolve dynamically over time, and its applicability is limited in traffic scenarios that lack a priori spatial information. On the other hand, data-driven methods based on attention mechanisms mitigate the reliance on graph structures, thus offering greater flexibility. However, such approaches typically treat nodes as an undifferentiated set [13], which restricts the model’s capacity to capture implicit spatial dependencies.

This paper suggests a Spatio-temporal Transformer Traffic Prediction Network Based on Multi-level Causal Attention (MLCAFormer) as a way to deal with the problems listed above in a systematic way. This model’s goal is to make traffic flow predictions more accurate by using a elaborate architectural design. The main things this study adds to the field are as follows:

1. We have developed a traffic prediction model, MLCAFormer, which integrates rich, multi-dimensional input data. Based entirely on attention mechanisms, it effectively captures both long- and short-term temporal dependencies as well as complex spatial correlations.
2. We propose a novel multi-level causal attention (MLCA) architecture. This architecture adopts a hierarchical design, partitioning attention windows of varying sizes at different levels to progressively fuse temporal information from a local to a global scale. This design enables the model to efficiently capture the long- and short-term dynamic dependencies in traffic flow. Additionally, we employ a masking operation in each attention window to ensure strict adherence to temporal causality.
3. We have designed a node-identity-aware spatial attention mechanism. While adopting a data-driven attention approach, we enhance the model’s ability to differentiate between nodes by assigning a unique identity embedding to each one. This helps the model to better capture traffic patterns among nodes and thereby adaptively learn the spatial correlations within the traffic network.
4. Comprehensive experiments were conducted on four widely-used real-world traffic datasets. The results show that MLCAFormer outperforms existing baseline models under various prediction settings. Further ablation studies also validate the effectiveness and necessity of each proposed component.

## Related work

### Traffic flow prediction and data interpolation methods

Traffic flow prediction is a topic of major interest because of its important social and economic benefits. Researchers in the past mostly used statistical models like Historical Average (HA) [14], Autoregressive Integrated Moving Average (ARIMA) [4], and Vector Autoregression (VAR) [15]. These kinds of models work best with linear data that doesn’t change much and is pretty consistent. But they don’t work as well with traffic data that isn’t linear and is more complicated. When these models have to deal with modern transportation networks that are getting bigger and bigger, they can’t make as many accurate predictions.

To adapt to traffic data with non-linear dependencies, subsequent researchers turned to machine learning methods. Some of the models used in this field are Random Forest [16], XGBoost [17], and Support Vector Regression (SVR) [18]. However, these machine learning-based methods usually need a lot of tedious feature engineering and a lot of complete data, which makes it hard to use them in real-world traffic networks.Researchers later looked into methods such as CNN and LSTM for traffic prediction to cut down on the need for feature engineering [19,20].

To further realize automated modeling and improve prediction efficiency, some research has begun to explore model architectures that surpass traditional recurrent and convolutional neural networks. Stochastic Configuration Networks (SCNs) are a typical example [21]. This model adopts an adaptive incremental construction strategy: the network begins with a single hidden node and progressively adds new ones automatically according to the complexity of the traffic flow data. Furthermore, it discards the conventional backpropagation algorithm, instead analytically calculating the output weights directly through the least squares method and pseudoinverse operations. This design addresses problems in traditional deep models, such as low training efficiency and an over-reliance on manual parameter tuning, providing a new technical approach for efficient traffic prediction.

The rise of deep learning has propelled a research paradigm shift toward “end-to-end” automated feature learning. Against this backdrop, an important branch of research utilizes tensor or matrix decomposition to preserve the multidimensional structure of traffic data, an approach that is particularly effective for handling missing data issues. For instance, Chen employed Tensor Completion techniques to recover spatio-temporal patterns from highly sparse data to perform data imputation [22,23]; other studies have constructed Latent Factor Analysis models to cope with extremely sparse scenarios where the missing data rate exceeds 90% [24]. Furthermore, these decomposition concepts have been extended to prediction tasks. Lin proposed the integration of tensor decomposition with 3D convolutional networks, enabling the model to directly predict future traffic conditions from incomplete data [25]. Notably, these models commonly incorporate a Temporal Regularized Constraint. By capturing the temporal dependencies in the data, this constraint can significantly enhance the accuracy of data imputation or prediction under sparse conditions.

Although decomposition-based methods provide valuable insights into dealing with incomplete data and understanding its internal structure, this paper focuses on another mainstream paradigm, the deep sequence model. This paradigm usually assumes that the data has been effectively preprocessed, so its research focus shifts to the design of the deep model architecture. At present, research under this paradigm is mainly carried out along two technical paths: first, using graph neural networks (GNNs) to explicitly encode the spatial topology of the transportation network; second, implicitly learning complex spatiotemporal dependencies from the data through the Transformer architecture.

### Spatio-temporal graph neural networks

A transportation network can be modeled as a graph where road sensors represent nodes and the roads form the edges. Graph Neural Networks (GNNs) are now widely used to predict traffic flow thanks to this graph-based representation. This line of research started with pioneering works like STGCN [7] and DCRNN [26]. STGCN captures spatio-temporal features by combining graph and temporal convolutions, while DCRNN models traffic flow as a diffusion process on the graph.

Later studies have built on this model, mostly by improving graph convolution operations. For example, STSGCN [27] uses a spatio-temporal synchronous graph to keep information from being lost, and AGCRN [28] learns node-specific parameters to find unique local patterns. Even though these methods have come a long way, they still have a major problem: they depend on a pre-defined adjacency matrix. This reliance creates two critical problems: first, physical proximity may not reflect true traffic correlations; second, a static graph cannot adapt to dynamic network variations caused by real-time events. To overcome this limitation, Graph WaveNet introduced an adaptive adjacency matrix capable of learning latent spatial dependencies directly from data. Taking this a step further, Lan et al. introduced the Dynamic Spatial-Temporal Aware Graph Neural Network (DSTAGNN) [29], which generates a data-driven dynamic graph by calculating the ’Spatial-Temporal Aware Distance’ (STAD) from historical traffic data.

This field has seen continuous development in recent years, with a growing diversity of research further enhancing the modeling capabilities of spatio-temporal graph networks. Auto-DSTSGN innovatively integrates dilated convolution into spatio-temporal synchronous graphs to efficiently capture long- and short-term dependencies, while also pioneering an automatic graph structure search mechanism that allows the model to flexibly construct and optimize graphs for different data scenarios. Other studies focus on real-time changes in graph topology; for instance, DGCRN utilizes a hyper-network at each RNN time step to dynamically generate a graph structure and combines it with a static graph [30]. In terms of node modeling, TPGNN draws inspiration from Personalized PageRank, enabling nodes to dynamically balance the influence of their own historical patterns with information from neighbors [31]. Furthermore, STPGNN addresses node heterogeneity by automatically identifying “pivotal nodes” in the network that possess strong traffic aggregation and distribution capabilities. It then employs a specialized pivotal graph convolution to precisely capture their unique spatio-temporal dependencies, thereby deepening the modeling of the network’s core areas [32].

### Attention mechanism and transformer

Another area of research utilizes the self-attention mechanism to learn spatio-temporal dependencies directly from data, thus reducing the reliance on pre-defined graph structures to some extent. However, these methods often do not consider the different identities of nodes, as they treat them as a single set. This makes it more difficult for the model to learn personalized traffic patterns. Recent research has begun to explore this issue. For instance, STD-MAE [33] introduces a two-dimensional spatio-temporal positional encoding, where node-specific information is mathematically generated based on each node’s unique index and integrated into the model’s input embeddings. Other methods use probabilistic models, such as variational Bayesian inference [34], in encoder-decoder frameworks to handle data uncertainty and learn more stable representations. Nevertheless, how to enable a model to distinguish node identities and learn personalized patterns using purely data-driven methods, without a pre-defined graph, remains a key question.

The Transformer architecture [8] marked a significant breakthrough in modeling the temporal dimension. Its ability to directly compute the dependency between any two points in a sequence makes it highly effective for capturing long-range dependencies.

The standard Transformer architecture was originally designed for processing natural language, but researchers have made several modifications to adapt it for traffic time-series data. Traffic Transformer [35], for example, uses multiple temporal encoding schemes to capture the continuity and periodicity of traffic flow. Similarly, STGAFormer [36] added a gated temporal self-attention module to better capture subtle changes and sudden events, which improved the accuracy of long-term forecasting. Recent studies such as STAEformer [13] have proposed adaptive embedding mechanisms to capture spatiotemporal relationships simultaneously, and CCDSReFormer [37] uses a dual-stream attention mechanism combined with ResNet to enhance local dependency representation while keeping computational costs low.However, these models typically apply the attention mechanism within a single window, limiting their ability to capture and integrate the multi-scale temporal dependencies inherent in traffic data. Furthermore, a key challenge is to capture these multi-scale dependencies while strictly maintaining temporal causality and preventing future information leakage.

In addition to improvements to the Transformer attention mechanism itself, several different research directions have emerged in recent years, bringing innovations in input representation and learning paradigms respectively. For example, in the field of input representation, PatchTST [38] significantly enhances the effectiveness of long-term time series forecasting by segmenting time series into “patches” as model input and adopting a strategy of independently processing each channel in multivariate series. In terms of learning paradigms, ST-LLM [39] realizes the integration of large language models with traffic prediction by adapting pre-trained LLMs through partially frozen attention strategies and spatial-temporal embeddings, demonstrating how general-purpose language models can be effectively transferred to structured spatio-temporal forecasting tasks.

In summary, although existing research has made significant progress, achieving efficient multi-scale temporal modeling and a fine-grained awareness of spatial node identities within the model remains a key challenge. The work presented in this paper is undertaken to address this very challenge.

## Problem formulation

### Problem definition

In this study, we define the traffic flow features across all sensors in the road network at a specific time *t* as a matrix $X_{t}\in {\mathbb{R}}^{N\times d_{in}}$, where *N* is the number of spatial nodes (sensors) and *d*_*in*_ is the dimension of the input features (e.g., traffic flow, speed). Consequently, the traffic data over a historical period can be represented as a three-dimensional tensor ${𝒳}_{1:P}=[X_{1},\ldots ,X_{P}]\in {\mathbb{R}}^{P\times N\times d_{in}}$, organized by the dimensions of time, space, and features, respectively.

The primary objective of traffic flow forecasting is to learn a mapping function *f* from historical observations to anticipate future traffic conditions. Specifically, the function leverages historical data spanning *P* previous time steps to forecast traffic conditions for the next *Q* time steps. This is mathematically formulated as:

$$\left[X_{t+1},\ldots ,X_{t+Q}]=f(X_{t-P+1:t}\right)$$ (1)

The key symbols and their interpretations used throughout this paper are summarized in Table 1.

**Table 1:** Symbols and interpretations.

Symbol	Interpretation
*N*	Total number of sensors (nodes) in the traffic network.
*P*	Length of the input sequence (observation window).
*Q*	Length of the output sequence (prediction horizon).
*d*_*in*_	Dimension of input features.
$d_{f},d_{h},d_{a}$	Dimensions of hidden features within the model.
$X\in {\mathbb{R}}^{P\times N\times d_{in}}$	Input tensor containing historical traffic data.
$\hat{Y}\in {\mathbb{R}}^{Q\times N\times 1}$	Predicted future traffic flow/speed.
*E*_*r*_	Embedding for raw traffic data.
*E*_*p*_	Embedding for periodic temporal features.
*E*_*c*_	Collaborative spatio-temporal embedding.
*E*_*id*_	Node identity embedding.

## Materials and methods

**Fig 1:**
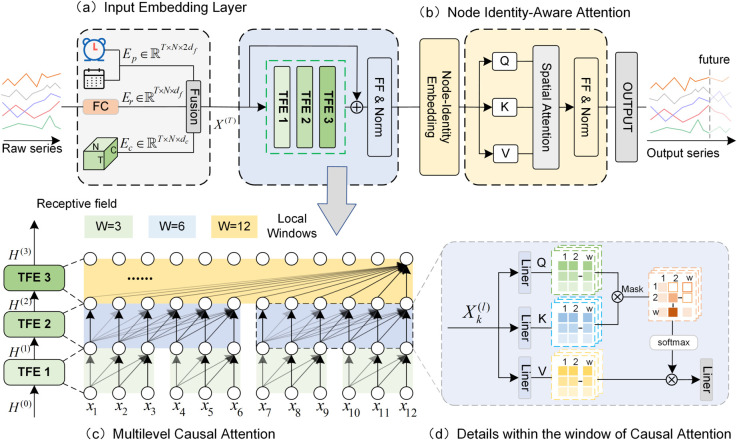
Overall architecture of the proposed MLCAFormer model. (a) Input embedding layer. (b) Node-identity-aware spatial attention. (c) Multi-level causal attention. (d) Details within the window of causal attention.

This paper proposes a framework for spatio-temporal traffic prediction, named MLCAFormer. As illustrated in Fig 1, the MLCAFormer model is built upon a modular architecture, which primarily consists of four core components: (1) an input embedding layer, designed to construct a comprehensive input representation by fusing raw data features, periodic temporal information, and a learnable collaborative spatio-temporal embedding; (2) a multi-level causal attention (MLCA) module, for efficiently capturing dynamic dependencies in the time series while strictly adhering to causality; (3) a node-identity-aware spatial attention module, which models dynamic spatial relationships by injecting an identity encoding for each node and leveraging a spatial attention mechanism; and (4) an output layer, responsible for mapping the final spatio-temporal feature representation to the multi-step prediction sequence. The detailed design of each module will be elaborated in the subsequent sections.

### Input embedding layer

In the input embedding layer, we integrate multi-dimensional input data, which mainly consists of three parts: original data embedding (*E*_*r*_), periodic embedding (*E*_*p*_), and collaborative spatio-temporal embedding (*E*_*c*_).

**Original data embedding.** To efficiently preserve essential characteristics of traffic information, we implement a fully-connected network to create original data embeddings, following the approach in [13]. These embeddings, denoted as $E_{r}\in {\mathbb{R}}^{P\times N\times d_{f}}$ where *d*_*f*_ represents hidden layer dimensionality, are computed through:

$$E_{r}=\text{FC}(X_{t-P+1:t})$$ (2)

Here, the fully-connected network FC(·) processes the input sequence *X*_*t*−*P* + 1:*t*_, mapping it into a feature embedding. This transformation preserves the core properties of the original data and extracts critical information, enabling the model to utilize the data more profoundly.

**Temporal periodic embedding.** We define two learning embedding matrices: the periodic day embedding $\Omega_{d}\in {\mathbb{R}}^{7\times d_{f}}$ and the time period embedding $\Omega_{t}\in {\mathbb{R}}^{m\times d_{f}}$, where 7 represents the number of days in a week, *m* represents the number of time segments in a day (in our experiment, we set *m* = 288 with a 5-minute interval), and *d*_*f*_ is the embedding dimension. Given a time series dataset with a collection of time points $\{\tau_{i}{\}}_{i=t-P+1}^{t}$ (where *P* is the length of the observation window), the daily index $D_{\tau }\in {\mathbb{R}}^{P}$ and the intra-day time index ${𝒥}_{\tau }\in {\mathbb{R}}^{P}$ corresponding to each time point can be extracted. By performing a table lookup operation, we obtain the corresponding embedding vectors $E_{d}\in {\mathbb{R}}^{P\times d_{f}}$ and $E_{t}\in {\mathbb{R}}^{P\times d_{f}}$. To create a comprehensive periodic representation, we first concatenate these two embedding vectors along the feature dimension. This operation yields a unified temporal embedding tensor, $E_{cat}\in {\mathbb{R}}^{P\times 2d_{f}}$, which simultaneously captures both weekly and daily patterns for each timestep. Since these periodic patterns are shared across all spatial locations, we then broadcast this purely temporal embedding to all *N* nodes. This is achieved by replicating the *E*_*cat*_ tensor *N* times—once for each spatial node—thereby creating the final temporal periodic embedding $E_{p}\in {\mathbb{R}}^{P\times N\times 2d_{f}}$.

**Collaborative spatio-temporal embedding.** Traffic data contains not only explicit time-series dependencies but also implicit spatio-temporal coupling characteristics determined by factors such as network topology, which cannot be directly observed. To model these latent dynamic relationships, this paper introduces a Collaborative Spatio-temporal Embedding, $E_{c}\in {\mathbb{R}}^{P\times N\times d_{c}}$. Instead of being computed directly from the data, it is a learnable parameter tensor that is optimized end-to-end during training. Its core function is to act as a “learnable spatio-temporal canvas,” supporting the subsequent encoder modules in more effectively and adaptively learning temporal evolutionary patterns and spatial interdependencies. This parameter is initialized using a Xavier uniform distribution.

Finally, we concatenate the three to obtain the input $X=E_{r}∥E_{p}∥E_{c}$ of the temporal encoding layer, where ∥ denotes the concatenation operation along the feature dimension. We denote $d_{h}=d_{f}+2d_{f}+d_{c}$, then $X\in {\mathbb{R}}^{P\times N\times d_{h}}$.

### Multi-level temporal causal attention

We propose a multi-level temporal causal attention (MLCA) mechanism. As shown in Fig 1(c), it is primarily composed of two parts: intra-layer window partitioning with attention calculation, and multi-scale feature fusion with inter-layer progression. Through a hierarchical design that partitions the sequence into non-overlapping attention windows of varying sizes at different layers, MLCA can systematically fuse temporal information from local to global scales.

**Intra-layer window division and attention calculation.** The MLCA module consists of *L* = 3 layers, each containing a temporal feature extractor (TFE). Within each layer *l*, we first partition the input sequence. Given an input sequence $H^{\left(l-1\right)}\in {\mathbb{R}}^{P\times N\times d_{h}}$(where *P* is the time step, *N* is the number of spatial nodes, and *d*_*h*_ denotes the feature dimension), the time window size for layer *l* is defined as

$${𝒲}_{l}=\left\lfloor \frac{P}{2^{L-l}}\right\rfloor$$ (3)

Taking *P* = 12 as an example, the window sizes from the bottom to the top layer are ${𝒲}_{1}=3,{𝒲}_{2}=6,{𝒲}_{3}=12$. Through the partitioning operation, the input sequence is reorganized as:

$$X^{\left(l\right)}=Partition(H^{\left(l-1\right)},{𝒲}_{l})\in {\mathbb{R}}^{\lceil P/{𝒲}_{l}\rceil \times {𝒲}_{l}\times N\times d_{h}}$$ (4)

Let $U=\lceil P/{𝒲}_{l}\rceil$ be the number of windows, and for each window $X_{k}^{\left(l\right)}\in {\mathbb{R}}^{{𝒲}_{l}\times N\times d_{h}}$, where k∈{0,1,…,U−1}, we generate the query, key, and value matrices via linear projections:

$$Q_{k}^{\left(l\right)}=X_{k}^{\left(l\right)}W_{q}^{\left(l\right)},K_{k}^{\left(l\right)}=X_{k}^{\left(l\right)}W_{k}^{\left(l\right)},V_{k}^{\left(l\right)}=X_{k}^{\left(l\right)}W_{v}^{\left(l\right)}$$ (5)

where $W_{q}^{\left(l\right)},W_{k}^{\left(l\right)},W_{v}^{\left(l\right)}\in {\mathbb{R}}^{d_{h}\times d_{h}}$ are learnable parameters. To ensure temporal causality, a causal mask matrix $M^{\prime }$ is introduced:

$$M_{i,j}^{\prime }=\left\{ \begin{array}{cc} 0 & \text{if }i\geq j,\\ -\infty & \text{otherwise}. \end{array}\right.$$ (6)

This mask ensures that each time step can only attend to the current and previous time steps. The attention output is then computed under this causal constraint:

$$Z_{k}^{\left(l\right)}=\text{Softmax}\left(\frac{Q_{k}^{\left(l\right)}(K_{k}^{\left(l\right)}{)}^{⊤}}{\sqrt{d_{h}}}+M^{\prime }\right)V_{k}^{\left(l\right)}$$ (7)

The outputs from each attention window are concatenated to form the output of the current layer $Z^{\left(l\right)}\in {\mathbb{R}}^{P\times N\times d_{h}}$:

$$Z^{\left(l\right)}=Concat(Z_{0}^{\left(l\right)},Z_{1}^{\left(l\right)},\ldots ,Z_{U-1}^{\left(l\right)})$$ (8)

**Multi-scale feature fusion and inter-layer progression.** The MLCA module implements a bottom-up fusion of multi-scale features through a cascaded structure. The output of the previous layer, *H*^(*l*−1)^, serves directly as the input for the current layer (*H*^(0)^ = *X*). The TFE in each layer first computes the attention features *Z*^(*l*)^:

$$Z^{\left(l\right)}={\text{TFE}}_{l}\left(H^{\left(l-1\right)}\right)$$ (9)

Subsequently, the output is processed through a sequence of residual connections, Layer Normalization, and a Feed-Forward Network (FFN) to generate the final output of the current layer, as described by the following procedure which accurately reflects the code implementation:

$$Y^{\left(l\right)}=Z^{\left(l\right)}+H^{\left(l-1\right)}$$ (10)

$$H^{\left(l\right)}=\text{FeedForward}(\text{LayerNorm}(Y^{\left(l\right)}))+Y^{\left(l\right)}$$ (11)

This output *H*^(*l*)^ is then propagated to the subsequent layer as its input.

Through this progressive layer-wise information transmission, the temporal receptive field gradually expands, enabling the effective capture of multi-scale temporal dependencies. The hierarchical structure allows the model to simultaneously attend to local details and global trends: the lower layer focuses on short-term fluctuations (${𝒲}_{1}=3$), the middle layer captures medium-term variations (${𝒲}_{2}=6$), and the top layer models long-term trends (${𝒲}_{3}=12$). After three layers, the output $H^{\left(3\right)}\in {\mathbb{R}}^{P\times N\times d_{h}}$ integrates rich features across multiple temporal scales, providing comprehensive information for prediction.

### Node-identity-aware spatial attention

**Fig 2:**
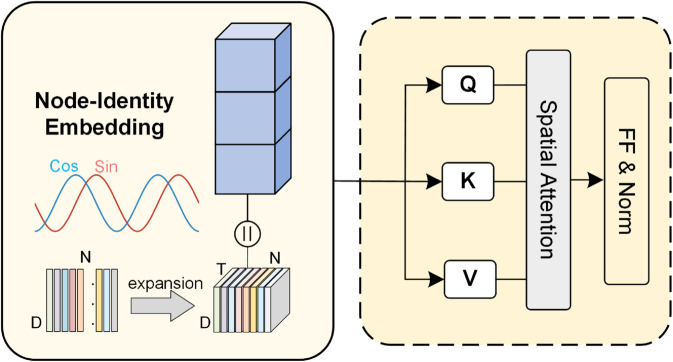
Node-identity-aware spatial attention module.

This paper proposes a node-identity-aware spatial attention mechanism. As illustrated in Fig 2, the mechanism comprises two key components: node identity embedding and spatial correlation computation. The former assigns a unique identity encoding to each node, while the latter is responsible for capturing deep spatial dependencies among nodes.

**Node identity embedding.** The core of this mechanism is to generate a deterministic and unique *identity vector*, $e_{i}\in {\mathbb{R}}^{d_{f}}$, for each node i∈{0,1,…,N−1} in the road network. To achieve this, we define an identity encoding function, Φ(·), which maps the discrete index of a node to a high-dimensional continuous vector representation through modulation with sine and cosine functions of different frequencies.

Specifically, the identity vector $e_{i}=\Phi (i)$ for node *i* is computed as follows, where $(e_{i}{)}_{j}$ is its *j*-th dimension:

$$({𝐞}_{i}{)}_{j}=\Phi (i{)}_{j}=\left\{ \begin{array}{cc} \sin\left(\frac{i}{10000^{\frac{j}{d_{f}}}}\right), & \text{if }j\text{ is even},\\ \cos\left(\frac{i}{10000^{\frac{j}{d_{f}}}}\right), & \text{if }j\text{ is odd}. \end{array}\right.$$ (12)

where *i* is the node index, and $j\in \{0,1,\ldots ,d_{f}-1\}$ is the dimension index of the identity vector.

By stacking the identity vectors $e_{0},e_{1},\ldots ,e_{N-1}$ along the node dimension, we form the complete node identity embedding matrix $E_{id}\in {\mathbb{R}}^{N\times d_{f}}$. To integrate this static node identity information with dynamic time-series features, we first broadcast the identity embedding matrix *E*_*id*_ along the time dimension (i.e., the input sequence length), generating a spatio-temporal identity representation ${\tilde{E}}_{id}\in {\mathbb{R}}^{P\times N\times d_{f}}$.

Next, this embedding containing node identity information, ${\tilde{E}}_{id}$, is concatenated with the output of the temporal encoding layer $H^{\left(L\right)}\in {\mathbb{R}}^{P\times N\times d_{h}}$ to obtain the final input *X*^(*S*)^ for the spatial attention module:

$$X^{\left(S\right)}=H^{\left(L\right)}∥{\tilde{E}}_{id}$$ (13)

where $X^{\left(S\right)}\in {\mathbb{R}}^{P\times N\times d_{a}}$, $d_{a}=d_{h}+d_{f}$. At this point, the tensor *X*^(*S*)^ not only carries rich dynamic temporal dependency information but also has an identity marker for each node.

**Spatial correlation computation.** This paper computes inter-node spatial correlations via a spatial self-attention mechanism. Specifically, the query, key, and value matrices are first obtained via linear transformations:

$$Q^{\left(S\right)}=X^{\left(S\right)}W_{q}^{\left(S\right)},K^{\left(S\right)}=X^{\left(S\right)}W_{k}^{\left(S\right)},V^{\left(S\right)}=X^{\left(S\right)}W_{v}^{\left(S\right)}$$ (14)

where $W_{q}^{\left(S\right)},W_{k}^{\left(S\right)},W_{v}^{\left(S\right)}\in {\mathbb{R}}^{d_{a}\times d_{a}}$ represents learnable parameters. Subsequently, the attention scores are computed as follows:

$$A^{\left(S\right)}=\text{Softmax}\left(\frac{Q^{\left(S\right)}K^{(S{)}^{𝖳}}}{\sqrt{d_{a}}}\right)$$ (15)

Here, $A^{\left(S\right)}\in {\mathbb{R}}^{P\times N\times N}$ captures the spatial relationships among nodes at different time steps, with each element representing the strength of the interaction between a pair of nodes. Using the computed attention weights, the weighted feature representation is obtained as:

$$Z^{\left(S\right)}=A^{\left(S\right)}V^{\left(S\right)}$$ (16)

where $Z^{\left(S\right)}\in {\mathbb{R}}^{P\times N\times d_{a}}$. It is worth noting that normalization, residual connections, and a multi-head attention mechanism are also applied, resulting in the final output of the spatiotemporal encoding layer, denoted as $Z^{\prime }$.

### Output layer

Finally, after obtaining the deep feature representation $Z^{\prime }\in {\mathbb{R}}^{P\times N\times d_{a}}$ from the spatio-temporal encoding layers, we employ a projection strategy to generate the final multi-step predictions. The core idea is to utilize the complete encoded sequence for each node to forecast its future values.

Specifically, the process is as follows: first, for each node, we flatten the feature representations across the entire input sequence length (*P*) and the hidden dimension (*d*_*a*_) into a single vector. This reshaped tensor is then fed into a single fully-connected (FC) layer, which directly projects this high-dimensional vector to a vector representing the desired prediction horizon of length *Q*. This can be expressed as:

$$\hat{Y}=\text{FC}(\text{Flatten}(Z^{\prime }))$$ (17)

where the Flatten(·) operation reshapes the tensor from ${\mathbb{R}}^{P\times N\times d_{a}}$ to ${\mathbb{R}}^{N\times (P\cdot d_{a})}$, and the FC layer maps it to ${\mathbb{R}}^{N\times Q}$. The output is then reshaped to the final prediction format $\hat{Y}\in {\mathbb{R}}^{Q\times N\times 1}$, where *Q* is the number of future time steps to be predicted. In our implementation, we predict only one feature dimension (traffic speed/flow), so the third dimension is 1.

## Experiments

### Experimental dataset

We comprehensively evaluated the MLCAFormer model on four real traffic datasets, testing traffic speed data (METR-LA, PEMS-BAY) and traffic flow data (PEMS04, PEMS08). Specific statistical features are detailed in Table 2.

**Table 2:** Dataset description.

Datasets	Nodes	Time steps	Sample rate
METR-LA	207	34272	5 min
PEMS-BAY	325	52116	5 min
PEMS04	307	16992	5 min
PEMS08	170	17856	5 min

### Experimental settings

**Implementation environment:** All experiments were conducted using PyTorch 1.11.0 on systems equipped with NVIDIA RTX 4090 GPUs. Dataset splits were 7:1:2 for training, validation, and testing on METR-LA and PEMS-BAY, and 6:2:2 on PEMS04 and PEMS08.These split ratios were chosen to align with common practices in prior literature using these benchmark datasets, ensuring a fair comparison.

**Hyperparameter settings:** The hyperparameters in this study were determined through experimental analysis. The feature embedding dimension (*d*_*f*_) and the adaptive embedding dimension (*d*_*c*_) were set to 24 and 80, respectively. The model’s architecture stacks 3 temporal encoding layers and 3 spatial encoding layers in sequence, with each attention mechanism equipped with 8 attention heads. The input and prediction windows are both set to 1 hour (i.e., *P* = 12 and *Q* = 12 time steps), with each step being 5 minutes apart. For the multi-scale causal attention module with an input length of 12, the window sizes for its three internal layers are [3, 6, 12].

**Optimization strategy:** For model training, we employed the Adam optimization algorithm with an initial learning rate of 0.001. A MultiStepLR scheduler was used to adjust the learning rate during the training process. The batch size was set to 16. To prevent overfitting and improve training efficiency, we implemented an early stopping mechanism with a patience of 30, which terminates training if the validation error does not improve for 30 consecutive epochs.

### Evaluation metrics

For assessing our model’s prediction capabilities, we utilize a combination of three standard evaluation indicators: MAE (Mean Absolute Error), MAPE (Mean Absolute Percentage Error), and RMSE (Root Mean Squared Error).

MAE (Mean Absolute Error) measures the average absolute deviation between predicted values and actual observations:

$$MAE=\frac{1}{n}\sum_{i=1}^{n}|{\hat{y}}_{i}-y_{i}|$$ (18)

MAPE (Mean Absolute Percentage Error) converts absolute errors into relative percentages, facilitating comparative analysis across datasets with different scales (requires non-zero actual values):

$$MAPE=\frac{1}{n}\sum_{i=1}^{n}\left|\frac{{\hat{y}}_{i}-y_{i}}{y_{i}}\right|\times 100\%$$ (19)

RMSE (Root Mean Square Error) emphasizes larger errors through squaring operations, reflecting the prediction result’s sensitivity to volatility:

$$RMSE=\sqrt{\frac{1}{n}\sum_{i=1}^{n}({\hat{y}}_{i}-y_{i}{)}^{2}}$$ (20)

Where *y*_*i*_ represents the actual traffic observation at time point *i*, ${\hat{y}}_{i}$ is the corresponding predicted value, and *n* is the total number of samples.

### Baseline models

In order to comprehensively evaluate the model we propose, this study selected 13 classic and cutting-edge traffic prediction methods as benchmarks, as detailed below:

- **HI** [40]: A simple yet powerful baseline that uses the most recent values from the input sequence as the direct prediction for the future.
- **GWNet** [41]: First applied Temporal Convolutional Network (TCN) to traffic prediction, and introduced skip connection mechanism to improve performance.
- **DCRNN** [26]: Combines Diffusion Convolutional Network with Recurrent Neural Network (RNN) for spatiotemporal modeling and prediction of traffic data.
- **AGCRN** [28]: Adaptively learns node-specific patterns, and infers interdependencies between different time periods.
- **STGCN** [7]: Integrates Graph Convolutional Network (GCN) with spatiotemporal convolution, effectively capturing spatiotemporal correlations in traffic data.
- **GTS** [42]: Proposes a new method that can jointly learn graph structures with Graph Neural Networks (GNN) while predicting multiple related time series, solving limitations of previous methods.
- **MTGNN** [43]: uses adaptive modules to capture both spatial and temporal dependencies while learning directional relationships between variables without the use of predefined graphs.
- **STNorm** [44]: Improves the interpretation of traffic patterns by extracting local features and high-frequency components from data using temporal and spatial normalization.
- **GMAN** [9]: Modifies influence weights between monitoring points to reflect time-varying dependencies in traffic systems by utilizing spatial and temporal attention mechanisms.
- **PDFormer** [10]: A traffic prediction model combining spatial self-attention mechanism and delay-aware module, effectively capturing dynamic spatial dependencies and information propagation delay, improving traffic flow prediction accuracy.
- **STID** [45]: To achieve effective prediction performance, temporal and spatial identity encoding is introduced using simple MLPs rather than intricate graph networks.
- **STAEformer** [13]: Combines spatiotemporal adaptive embedding with standard Transformer architecture, achieving outstanding prediction performance.
- **TASSGN** [46]: A model designed to tackle the graph indistinguishability problem by simultaneously learning the structural and semantic aspects of graphs and making them temporally aware through a self-sampling mechanism.

### Comparison and analysis

To comprehensively evaluate the overall performance of the MLCAFormer model, this section outlines a series of detailed experiments. First, the model’s performance is compared against 13 mainstream baselines on four public traffic datasets. Subsequently, systematic ablation studies and a hyperparameter sensitivity analysis are conducted to validate the effectiveness of the model’s individual components and the rationale behind its key parameter settings. Building on this foundation, we further investigate the model from three dimensions: adaptability, computational efficiency, and interpretability. This includes assessing its performance in various road scenarios, comparing the computational costs of different models, and conducting a visual case study to analyze its ability to learn spatio-temporal correlations.

**Discussion of baseline comparison.** We tested our MLCAFormer architecture on four real traffic datasets—two concentrating on traffic speed (METR-LA and PEMS-BAY) and two recording traffic volume (PEMS04 and PEMS08)—to confirm its effectiveness. Tables 3 and 4 show the results of our experiments; bolded values show the best performance, underlined values show the second-best results.

**Table 3:** Forecasting performance comparison on the METR-LA and PEMS-BAY datasets.

Dataset	Method	Horizon 3 (15 min)			Horizon 6 (30 min)			Horizon 12 (60 min)		
		MAE	RMSE	MAPE(%)	MAE	RMSE	MAPE(%)	MAE	RMSE	MAPE(%)
**METR-LA**	HI	6.80	14.21	16.72	6.80	14.21	16.72	6.80	14.20	10.15
	GWNet	2.69	5.15	6.99	3.08	6.20	8.47	3.51	7.28	9.96
	DCRNN	2.67	5.16	6.86	3.12	6.27	8.42	3.54	7.47	10.32
	AGCRN	2.85	5.53	7.63	3.20	6.52	9.00	3.59	7.45	10.47
	STGCN	2.75	5.29	7.10	3.15	6.35	8.62	3.60	7.43	10.35
	GTS	2.75	5.27	7.12	3.14	6.33	8.62	3.59	7.44	10.25
	MTGNN	2.69	5.16	6.89	3.05	6.13	8.16	3.47	7.21	9.70
	STNorm	2.81	5.57	7.40	3.18	6.59	8.47	3.57	7.51	10.24
	GMAN	2.80	5.55	7.41	3.12	6.49	8.73	3.44	7.35	10.07
	PDFormer	2.83	5.45	7.77	3.20	6.46	9.19	3.62	7.47	10.91
	STID	2.82	5.53	7.75	3.19	6.57	9.39	3.55	7.55	10.95
	STAEformer	2.65	5.11	6.85	2.97	6.00	8.13	3.34	7.02	9.70
	TASSGN	2.72	5.27	7.14	3.03	6.05	8.22	3.39	7.01	9.78
	MLCAFormer	**2.62**	**5.05**	**6.72**	**2.93**	**5.96**	**7.95**	**3.30**	**6.97**	**9.47**
PEMS-BAY	HI	3.06	7.05	6.85	3.06	7.04	6.84	3.05	7.03	6.83
	GWNet	1.30	**2.73**	2.71	1.63	3.73	3.73	1.99	4.60	4.71
	DCRNN	1.31	2.76	2.73	1.65	3.75	3.71	1.97	4.60	4.68
	AGCRN	1.35	2.88	2.91	1.67	3.82	3.81	1.94	4.50	4.55
	STGCN	1.36	2.88	2.86	1.70	3.84	3.79	2.02	4.63	4.72
	GTS	1.37	2.92	2.85	1.72	3.86	3.88	2.06	4.60	4.88
	MTGNN	1.33	2.80	2.81	1.66	3.77	3.75	1.95	4.50	4.62
	STNorm	1.33	2.82	2.76	1.65	3.77	3.66	1.92	4.45	4.46
	GMAN	1.35	2.90	2.87	1.65	3.82	3.74	1.91	4.49	4.52
	PDFormer	1.32	2.83	2.78	1.64	3.79	3.71	1.91	4.43	4.51
	STID	1.31	2.79	3.78	1.64	3.73	3.73	1.91	4.42	4.55
	STAEformer	1.31	2.78	2.76	1.62	3.68	3.62	1.88	4.34	4.41
	TASSGN	1.29	**2.73**	2.71	**1.59**	**3.60**	3.58	**1.86**	4.34	4.32
	MLCAFormer	**1.28**	**2.73**	**2.66**	**1.59**	3.63	**3.50**	**1.86**	**4.30**	**4.30**

**Table 4:** Forecasting performance comparison on the PEMS04 and PEMS08 datasets.

Model	PEMS04 (N=307)			PEMS08 (N=170)		
	MAE	RMSE	MAPE(%)	MAE	RMSE	MAPE(%)
HI	42.35	61.66	29.92	36.66	50.45	21.63
GWNet	18.53	29.92	12.89	14.40	23.39	9.21
DCRNN	19.63	31.26	13.59	15.22	24.17	10.21
AGCRN	19.38	31.25	13.40	15.32	24.41	10.03
STGCN	19.57	31.38	13.44	16.08	25.39	10.60
GTS	20.96	32.95	14.66	16.49	26.08	10.54
MTGNN	19.17	31.70	13.37	15.18	24.24	10.20
STNorm	18.96	30.98	12.69	15.41	24.77	9.76
GMAN	19.14	31.60	13.19	15.31	24.92	10.13
PDFormer	18.36	30.03	12.00	13.58	23.41	9.05
STID	18.38	29.95	12.04	14.21	23.28	9.27
STAEformer	18.22	30.18	11.98	13.46	23.25	8.88
TASSGN	**18.02**	**29.44**	12.44	13.75	22.97	9.19
MLCAFormer	18.26	30.15	**11.96**	**13.42**	**23.16**	**8.85**

The results of the experiments show that traditional statistical methods, like the Historical Average (HA), make bigger mistakes than the other methods that were tested. They aren’t doing well because they can’t handle complicated time dependencies and take spatial correlations into account. On the other hand, deep learning models based on spatio-temporal graph neural networks and the Transformer architecture did very well, proving that they are good at predicting traffic. The STID model also got good results, even though it used a relatively simple Multi-Layer Perceptron (MLP) architecture. The reason for this performance is that it adds spatio-temporal identity information to the input data, which makes it easier to tell apart spatio-temporal data.

Table 3 displays the comparative results for traffic speed prediction. We evaluated all models across three prediction horizons: 15 min, 30 min, and 60 min. The results indicate that on the METR-LA dataset, MLCAFormer demonstrates a prominent advantage, outperforming the other baseline models on MAE, RMSE, and MAPE metrics across all prediction horizons. Notably, for the 60-minute long-term forecast, its MAE, RMSE, and MAPE reached 3.30, 6.97, and 9.47%, respectively. On the PEMS-BAY dataset, MLCAFormer also delivered an outstanding performance. Although the competition with other models, such as TASSGN and GWNet, was very close on certain individual metrics, MLCAFormer maintained its leading position in overall performance.

Table 4 shows the performance of the various models on traffic flow prediction. On the PEMS04 dataset, TASSGN performs better on the MAE (18.02) and RMSE (29.44) metrics, whereas MLCAFormer achieves the best performance on the MAPE metric with a value of 11.96%. On the PEMS08 dataset, MLCAFormer secures the top performance across all metrics: an MAE of 13.42, an RMSE of 23.16, and a MAPE of 8.85%, outperforming the other baseline methods. These results demonstrate the model’s strong generalization performance.

MLCAFormer performs exceptionally well on the MAPE metric. Specifically, for the 60-minute prediction on the METR-LA dataset, MLCAFormer’s MAPE is 9.47%, which is a 3.2% and 2.4% improvement over TASSGN’s 9.78% and STAEformer’s 9.70%, respectively. On the PEMS-BAY dataset, its MAPE for the 60-minute prediction is 4.30%, showing a slight but consistent improvement over TASSGN’s 4.32%. This indicates that the model maintains high relative accuracy across different traffic flow conditions, delivering reliable predictions in both congested and uncongested traffic states.

As the prediction horizon gets longer, all of the models’ performance gets worse, which is what usually happens with forecasting tasks. MLCAFormer does a better job of modeling long-range temporal dependencies than other models because it has a smaller drop in performance when making long-term predictions. The proposed multi-level temporal causal attention mechanism is mostly responsible for this advantage because it can effectively capture and combine temporal dependencies from small to large scales.

**Fig 3:**
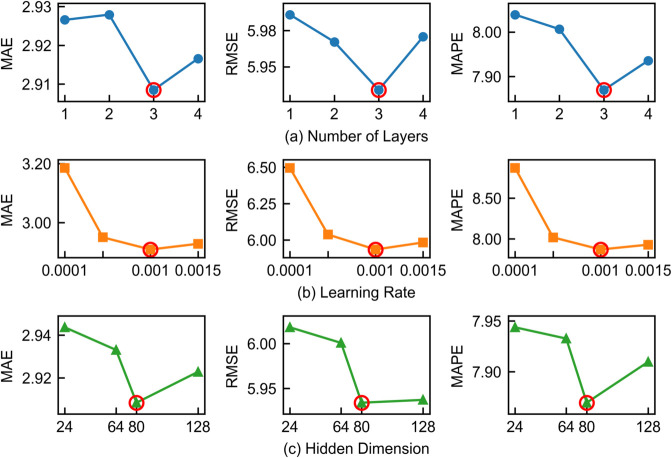
Sensitivity analysis of key hyperparameters on the METR-LA dataset. Performance variation with respect to (a) the number of MLCA layers, (b) the learning rate, and (c) the hidden dimension of the collaborative spatio-temporal embedding.

**Sensitivity analysis.** We did a full sensitivity analysis on three important parts of the MLCAFormer model using the METR-LA dataset to see how key hyperparameters affect model performance and to help us find the best configuration. The three parts we looked at were the number of layers in the multi-level causal attention (MLCA) module, the hidden dimension of the collaborative spatio-temporal embedding, and the learning rate used during training. Performance was evaluated using the MAE, RMSE, and MAPE metrics.To showcase the comprehensive prediction performance, these metrics were averaged over the 12-step prediction horizon. The results are shown in Fig 3

Impact of Layer Number.The number of layers in the MLCA module determines the depth and breadth of temporal feature extraction. We tested configurations with {1, 2, 3, 4} layers, with their corresponding hierarchical window sizes detailed in Table 5. As shown in Fig 3(a), the model achieves its best performance when the number of layers is set to 3 (corresponding to windows {3, 6, 12}). Fewer layers (1-2) perform worse due to an inability to effectively capture multi-granularity information, whereas increasing to 4 layers leads to a slight performance degradation, likely due to minor overfitting. Therefore, a 3-layer structure strikes an optimal balance between model capacity and generalization.

**Table 5:** Window size configurations for different layer counts.

Number of Layers	Window Sizes per Layer
1 Layer	{12}
2 Layers	{6, 12}
3 Layers	{3, 6, 12}
4 Layers	{1, 3, 6, 12}

Impact of Learning Rate.The learning rate is critical for determining the model’s convergence performance. As depicted in Fig 3(b), the model performs best when the learning rate is set to 0.001. A learning rate that is too low (0.0001) results in slow convergence and the poorest performance, while a rate that is too high (0.0015) can destabilize the training process and harm the final results.

Impact of Hidden Dimension. The hidden dimension (*d*_*c*_) of the Collaborative Spatio-temporal Embedding (*E*_*c*_) affects the complexity and precision with which the model adaptively learns spatio-temporal dependencies. As shown in Fig 3(c), we tested this dimension across values of {24, 64, 80, 128} and found that performance is optimal when *d*_*c*_ = 80. This indicates that lower dimensions limit the model’s parameter capacity, preventing it from fully learning complex dependency structures. Conversely, increasing the dimension to 128 yields no further improvement, suggesting that an 80-dimensional embedding space is sufficient to capture the key dependency patterns driving traffic flow dynamics, striking the best balance between model expressiveness and computational efficiency.

**Ablation study.** We conducted a series of ablation experiments to verify the effectiveness and contribution of each fundamental component in our proposed method. We conducted a comprehensive analysis and comparison of four critical variants of our methodology. The four variants are described in detail as follows:

1. MLCAFormer-NM: We removed the multi-level causal attention mechanism from the temporal encoding layer to evaluate its specific impact on model performance.
2. MLCAFormer-NN: Instead of utilizing the node-identity-aware spatial attention mechanism, we directly employed standard attention mechanisms to extract spatial features.
3. MLCAFormer-NP: In the multi-dimensional input embedding module, we removed the periodic embedding component, retaining only the traffic flow data embedding and collaborative spatio-temporal embedding, to verify the influence of periodic features on the model’s prediction performance.
4. MLCAFormer-NC: In the multi-dimensional input embedding module, we retained the basic traffic flow data embedding and periodic embedding, but removed the collaborative spatiotemporal embedding module, replacing it with conventional spatial embedding as in the STID study [45]. This comparative experiment aims to evaluate the contribution of the collaborative spatiotemporal embedding module to the overall prediction performance of the model.

Table 6 presents the results of our ablation studies in detail. The comparison of each variant’s performance across various evaluation metrics indicates that every component of our method enhances the model’s overall performance. From the experimental data and analyses, we conclude the following:

1. Removing the multi-level causal attention (MLCA) module resulted in the most significant performance degradation, with the MAE increasing by 2.1% and 1.9% on the METR-LA and PEMS04 datasets, respectively. This quantitatively confirms the ability of MLCA to effectively capture multi-scale temporal dependencies through its hierarchical structure.
2. The removal of the node-identity-aware spatial attention module also led to a performance decline, with the MAE on METR-LA increasing from 3.30 to 3.34. This validates our hypothesis that if the model cannot effectively distinguish node uniqueness during spatial attention computation, it will struggle to learn the personalized traffic patterns of individual nodes.
3. Periodic embedding plays a significant role in capturing daily, weekly, and other periodic patterns in traffic flow. Experimental results show that after removing periodic embedding, the model’s prediction ability for traffic scenarios with obvious temporal periodicity features declines, confirming the necessity of considering temporal periodicity in traffic flow prediction.
4. Similarly, removing the collaborative spatio-temporal embedding led to a significant performance decay (MAE increased by 2.1% on METR-LA). This validates the module’s core function as a “learnable spatio-temporal canvas”; without it, the model loses the ability to adaptively learn the data-driven, implicit spatio-temporal dependencies, leading to decreased performance. In summary, a rich input representation is fundamental to high-performance forecasting.

**Table 6:** Performance comparison of different model variants on the METR-LA and PEMS04 datasets.

Model Variant	METR-LA			PEMS04		
	MAE	RMSE	MAPE(%)	MAE	RMSE	MAPE(%)
MLCAFormer	**3.30**	**6.97**	**9.47**	**18.26**	**30.15**	**11.96**
MLCAFormer-NM	3.37	7.12	9.80	18.61	30.53	12.37
MLCAFormer-NN	3.34	7.05	9.68	18.36	30.50	12.13
MLCAFormer-NP	3.40	7.12	9.64	18.47	30.41	12.16
MLCAFormer-NC	3.37	7.10	9.65	18.63	30.54	12.31

**Fig 4:**
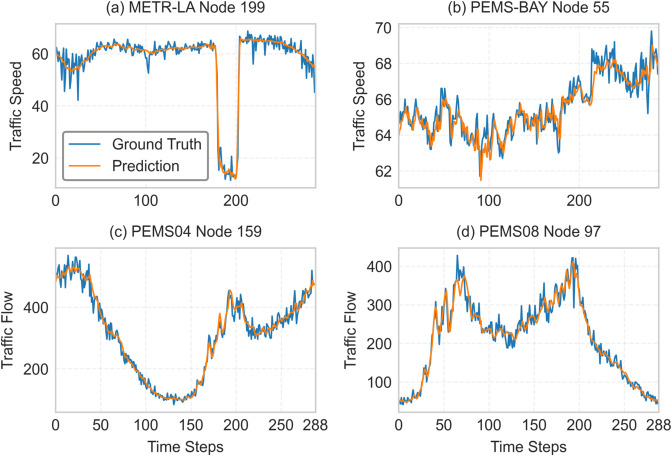
Visualization of node predictions on four datasets shows that the prediction curves of MLCAFormer are basically consistent with the actual curves. (a) METR-LA node 199. (b) PEMS-BAY node 55. (c) PEMS04 node 159. (d) PEMS08 node 97.

**Prediction visualization.** We chose one node at random from each of the four datasets for visualization in order to demonstrate the efficacy of our suggested model. A comparison of expected and actual values over a 24-hour period is shown in Fig 4. The figure demonstrates the model’s ability to accurately represent dynamic changes in traffic data by showing how closely the prediction results generated by our model match actual traffic flow patterns. Additionally, MLCAFormer shows a high degree of flexibility in responding to large-scale, short-term changes in traffic data. Fig 4(a) shows that our model maintains high prediction accuracy on the METR-LA dataset within the interval t∈[170,210].

**Fig 5:**
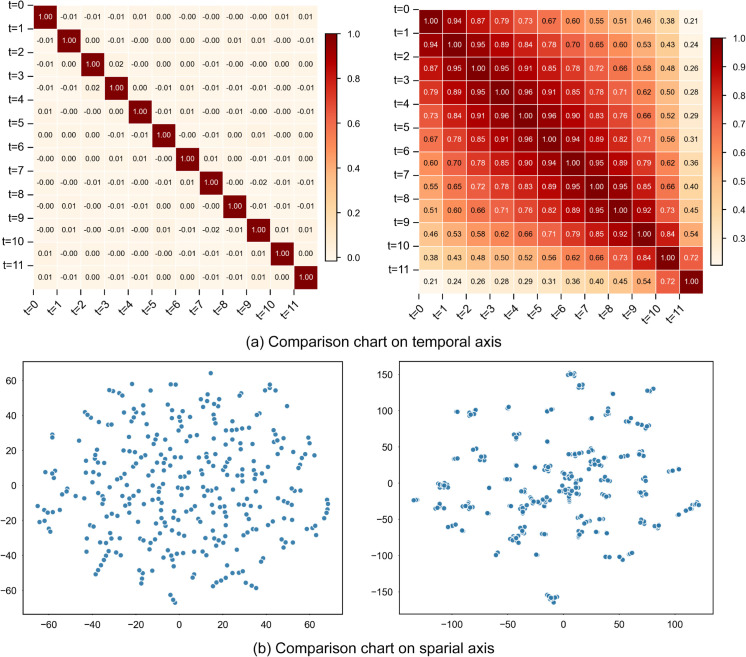
Visualization of the collaborative spatiotemporal embedding on the PEMS-BAY dataset, showing correlation patterns before and after model training. (a) Comparison chart on temporal axis. (b) Comparison chart on spatial axis.

**Visualization of the collaborative spatiotemporal embedding.** To further investigate the model’s ability to extract spatio-temporal correlations in data, we use the PEMS-BAY dataset as an example and show the visualization results of the collaborative spatio-temporal embedding(*E*_*c*_) along the time axis and space axis in Fig 5.

We computed correlation coefficients among 12 input frames in the temporal dimension and illustrated them as a heat map, as seen in Fig 5(a). The comparative study indicates that the trained model effectively captures intrinsic correlations between time steps, in contrast to the original random embedding. The heat map distinctly illustrates that each frame exhibits a strong association with neighboring frames, with correlation diminishing progressively as time intervals extend, effectively representing the long-term and short-term temporal interdependence inherent in traffic sequences.

In the spatial dimension, we employed T-SNE dimensionality reduction technique to generate Fig 5(b), which reveals the spatial associations among 307 sensor nodes in the PEMS-BAY dataset. The distance between nodes in the two-dimensional space reflects the strength of their correlation, with closer distances indicating stronger correlations. Experimental results show that before training, the nodes exhibited a randomly dispersed state without obvious clustering characteristics; whereas after training, the embedding representations of different nodes formed distinct clustering structures.

Visualization of the Collaborative Spatio-Temporal Embedding module (*E*_*c*_) demonstrates that our proposed model effectively learns the spatio-temporal dependencies within the traffic data. The integration of node identity embeddings with the spatial self-attention mechanism effectively enhances the model’s ability to differentiate between nodes, while the multi-level causal attention mechanism successfully establishes both long- and short-term temporal dependencies.

**Fig 6:**
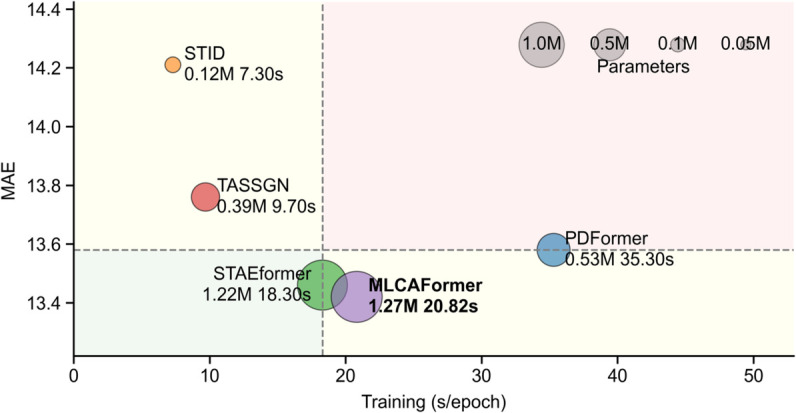
Trade-off between performance and efficiency. Comparison of MAE, training time per epoch, and the number of trainable parameters (indicated by bubble size) for different models on the PEMS08 dataset.

**Computation costs.** To comprehensively evaluate computational efficiency, we compare the number of trainable parameters and the average training time per epoch for our proposed MLCAFormer against four other baseline models on the PEMS08 dataset, using an NVIDIA RTX 4090 GPU. The results are visualized in Fig 6, where the dashed line indicates the median of the Mean Absolute Error (MAE) and the Training Time per Epoch across all models, which can be used to evaluate the relative position of each model in terms of performance and efficiency. The analysis reveals that, among the compared models, Transformer-based architectures generally require more parameters and longer training times than the MLP-based STID or the GNN-based TASSGN, which is attributable to the computational demands of the attention mechanism. However, this increased computational overhead also corresponded to an improvement in prediction accuracy.

An interesting finding is that parameter count and training time are not always positively correlated. For example, PDFormer (0.53M parameters) requires a longer training time per epoch than the larger MLCAFormer (1.27M parameters). Although our proposed MLCAFormer has a relatively large parameter count among the selected models, its training efficiency (20.82 s/epoch) is comparable to the high-performing STAEformer (18.30 s/epoch). Crucially, it achieves the lowest MAE (13.42), demonstrating the best prediction performance in this comparison. In summary, MLCAFormer strikes a balance between prediction accuracy and computational efficiency.

**Performance under different roads.** To investigate the model’s adaptability to different real-world traffic scenarios, we selected three representative roads from the PEMS04 dataset for analysis. These roads represent three typical traffic patterns: Road1 (stable traffic flow), Road2 (frequent fluctuations in traffic flow), and Road3 (traffic flow with both frequent and large-amplitude fluctuations). As shown in Table 7, we compiled the performance metrics for these three road types at 15-min, 30-min, and 60-min prediction horizons.

**Table 7:** Model performance on roads with different traffic patterns.

Road Type	15 min		30 min		60 min	
	MAE	RMSE	MAE	RMSE	MAE	RMSE
Road1 (Stable)	5.06	7.29	5.59	7.83	6.84	8.41
Road2 (Fluctuating)	13.82	19.51	16.17	23.02	17.84	24.01
Road3 (Volatile)	19.67	29.48	22.58	33.20	23.89	35.93

The data in the table show that the prediction accuracy is related to the stability of the road’s traffic flow itself; as fluctuations intensify, the prediction accuracy decreases accordingly, which aligns with the fundamental regularities of traffic forecasting. The data also indicates that from short-term to long-term predictions, the model’s error increases relatively slowly, suggesting that the model can adapt well from short-term to long-term forecasting.

**Fig 7:**
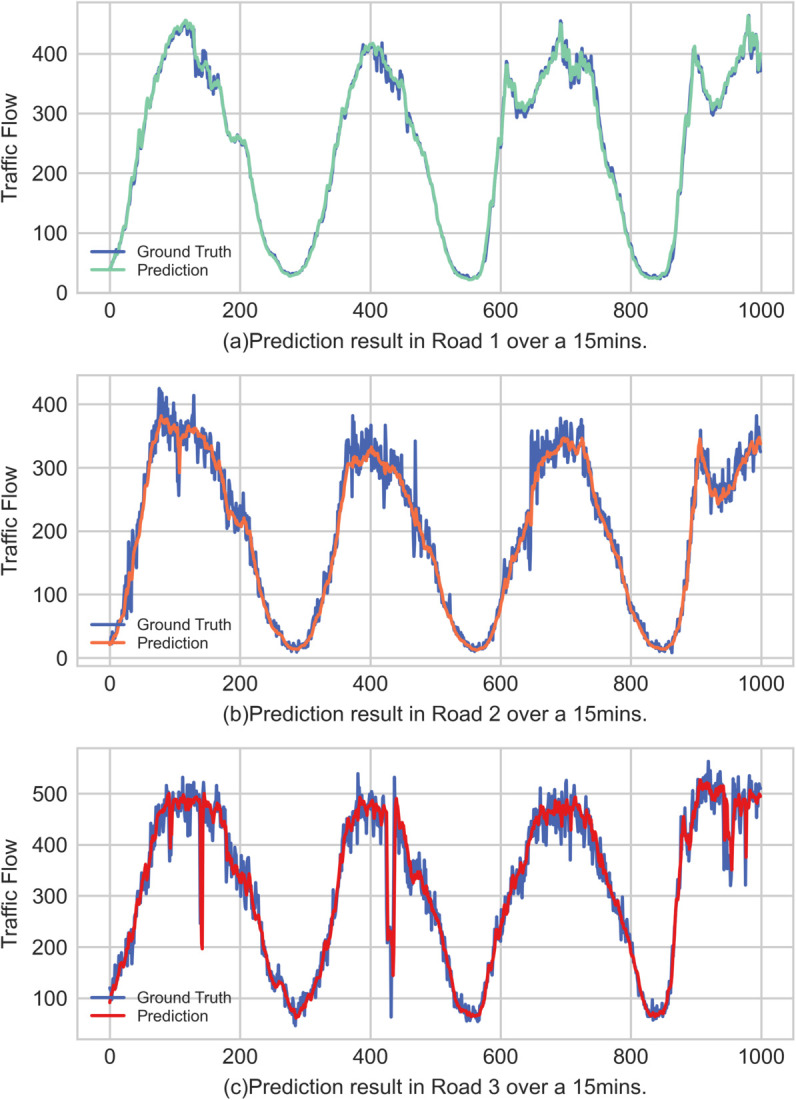
Visualization of 15-minute ahead prediction performance on three representative roads from the PEMS04 dataset.

Fig 7(a) displays the 15-minute prediction results for the three types of roads. As can be seen, for both stable and highly fluctuating roads, the predicted values fit the ground truth well. For example, in Fig 7(b), the predicted values are very close to the true values. Furthermore, as shown in Fig 7(c), our model accurately predicts the changing trends of the data and is able to capture the larger local fluctuations within the data.

**Fig 8:**
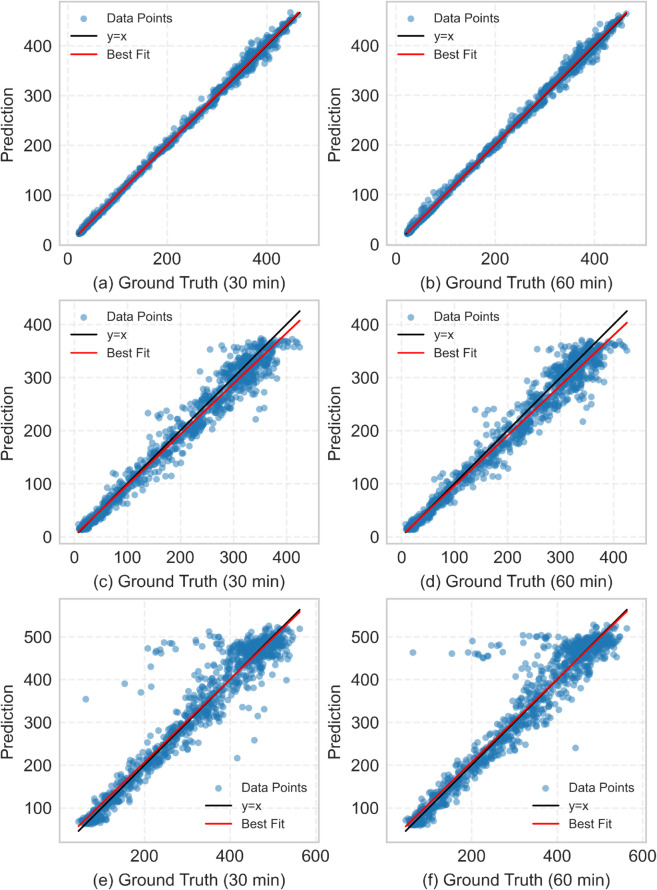
Scatter plots illustrating prediction accuracy for medium- and long-term horizons on different road types. The plots show the correlation between predicted values and ground truth for (a-b) Road 1, (c-d) Road 2, and (e-f) Road 3 at 30-minute and 60-minute prediction horizons. Points clustering along the y=x diagonal indicate high prediction accuracy.

Fig 8 shows the 30-min and 60-min prediction results for the three roads. As seen in the plots for the stable road, the model’s predictions are very close to the ground truth. For the roads with intense fluctuations, most data points remain close to the diagonal line (y=x), indicating that the model does not produce systematic bias and maintains good accuracy and stability in medium- to long-term predictions.

## Conclusion

In conclusion, to address the challenge of capturing complex long- and short-term temporal dependencies in traffic prediction tasks, and the issue of data-driven methods based on attention mechanisms struggling to effectively distinguish node identities, this paper proposes a Spatio-temporal Transformer traffic prediction network based on a multi-level causal attention mechanism (MLCAFormer), which has effectively improved prediction accuracy. The research conclusions are as follows:

In the input layer, the model integrates a multi-dimensional embedding module rich with information. Building on this foundation, its core multi-level causal attention (MLCA) mechanism effectively captures temporal dependencies from local to global scales through a hierarchical architecture and optimized intra-layer attention windows. Simultaneously, a node-identity-aware spatial attention mechanism assigns a unique identity encoding to each node through an embedding method, which enhances the model’s capability to capture spatial dependencies among road nodes. Comprehensive experimental results have verified the predictive accuracy of the model and have also demonstrated its adaptability across different types of roads.

Despite the excellent performance of MLCAFormer, this study still has certain limitations. On the one hand, the model’s computational cost is relatively high (in terms of the number of parameters and training time), which could limit its deployment in real-world road applications. On the other hand, actual traffic conditions often involve various sudden incidents, which the model does not take into consideration.

Future research can be dedicated to model lightweighting, incorporating external factors such as weather and public holidays into the model, and exploring effective prediction methods for scenarios with missing traffic data, in order to further enhance the model’s practicality and accuracy.
